# Image quality in whole-body MRI using the MY-RADS protocol in a prospective multi-centre multiple myeloma study

**DOI:** 10.1186/s13244-023-01498-3

**Published:** 2023-10-15

**Authors:** Sam Keaveney, Alina Dragan, Mihaela Rata, Matthew Blackledge, Erica Scurr, Jessica M. Winfield, Joshua Shur, Dow-Mu Koh, Nuria Porta, Antonio Candito, Alexander King, Winston Rennie, Suchi Gaba, Priya Suresh, Paul Malcolm, Amy Davis, Anjumara Nilak, Aarti Shah, Sanjay Gandhi, Mauro Albrizio, Arnold Drury, Guy Pratt, Gordon Cook, Sadie Roberts, Matthew Jenner, Sarah Brown, Martin Kaiser, Christina Messiou

**Affiliations:** 1 MRI Unit, The Royal Marsden NHS Foundation Trust, London, UK; 2 Division of Radiotherapy and Imaging, The Institute of Cancer Research, London, UK; 3 Clinical Trials and Statistics Unit, The Institute of Cancer Research, London, UK; 4 University Hospital Southampton NHS Foundation Trust, Southampton, UK; 5 University Hospitals of Leicester NHS Trust, Leicester, UK; 6 University Hospitals of North Midlands NHS Trust, Stoke-on-Trent, UK; 7 University Hospitals Plymouth NHS Trust, Plymouth, UK; 8 Norfolk & Norwich University Hospitals NHS Foundation Trust, Norwich, UK; 9 Epsom & St. Helier University Hospitals NHS Trust, Epsom, UK; 10 Worcestershire Acute Hospitals NHS Trust, Worcester, UK; 11 Hampshire Hospitals NHS Foundation Trust, Basingstoke, UK; 12 North Bristol NHS Trust, Bristol, UK; 13 Nottingham University Hospitals NHS Trust, Nottingham, UK; 14 Royal Bournemouth and Christchurch Hospitals NHS Foundation Trust, Bournemouth, UK; 15 University Hospitals Birmingham NHS Foundation Trust, Birmingham, UK; 16 Clinical Trials Research Unit, Leeds Institute of Clinical Trials Research, University of Leeds, Leeds, UK; 17 Leeds Cancer Centre, Leeds Teaching Hospitals NHS Trust, Leeds, UK

**Keywords:** Whole-body MRI, Myeloma, Multi-centre trial, Quality control

## Abstract

**Background:**

The Myeloma Response Assessment and Diagnosis System (MY-RADS) guidelines establish a standardised acquisition and analysis pipeline for whole-body MRI (WB-MRI) in patients with myeloma. This is the first study to assess image quality in a multi-centre prospective trial using MY-RADS.

**Methods:**

The cohort consisted of 121 examinations acquired across ten sites with a range of prior WB-MRI experience, three scanner manufacturers and two field strengths. Image quality was evaluated qualitatively by a radiologist and quantitatively using a semi-automated pipeline to quantify common artefacts and image quality issues. The intra- and inter-rater repeatability of qualitative and quantitative scoring was also assessed.

**Results:**

Qualitative radiological scoring found that the image quality was generally good, with 94% of examinations rated as good or excellent and only one examination rated as non-diagnostic. There was a significant correlation between radiological and quantitative scoring for most measures, and intra- and inter-rater repeatability were generally good.

When the quality of an overall examination was low, this was often due to low quality diffusion-weighted imaging (DWI), where signal to noise ratio (SNR), anterior thoracic signal loss and brain geometric distortion were found as significant predictors of examination quality.

**Conclusions:**

It is possible to successfully deliver a multi-centre WB-MRI study using the MY-RADS protocol involving scanners with a range of manufacturers, models and field strengths. Quantitative measures of image quality were developed and shown to be significantly correlated with radiological assessment. The SNR of DW images was identified as a significant factor affecting overall examination quality.

**Trial registration:**

ClinicalTrials.gov, NCT03188172, Registered on 15 June 2017.

**Critical relevance statement:**

Good overall image quality, assessed both qualitatively and quantitatively, can be achieved in a multi-centre whole-body MRI study using the MY-RADS guidelines.

**Key points:**

• A prospective multi-centre WB-MRI study using MY-RADS can be successfully delivered.

• Quantitative image quality metrics were developed and correlated with radiological assessment.

• SNR in DWI was identified as a significant predictor of quality, allowing for rapid quality adjustment.

**Graphical Abstract:**

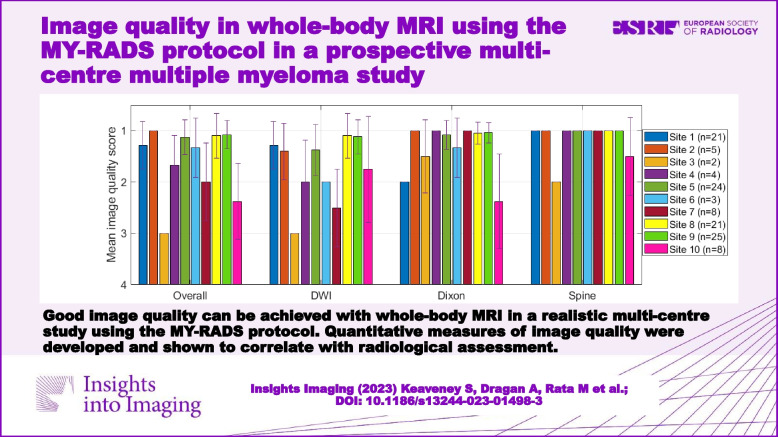

**Supplementary Information:**

The online version contains supplementary material available at 10.1186/s13244-023-01498-3.

## Background

Whole-body magnetic resonance imaging (WB-MRI) is a technique for imaging focal bone marrow lesions with superior sensitivity to ^18^F Fluorodeoxyglucose positron emission tomography/computed tomography (PET/CT) in patients with myeloma [1]. Contemporary WB-MRI is integral to international and national guidelines for patients with a suspected diagnosis of myeloma [2, 3]; however, it is not yet consistently available outside of centres with specialist expertise [2].

The need for standardised acquisition, interpretation and reporting of WB-MRI in myeloma led to the development of the Myeloma Response Assessment and Diagnosis System (MY-RADS) [4]. MY-RADS recommends key imaging parameters for WB diffusion weighted imaging (DWI), T_1_-weighted (T_1_w) Dixon imaging and T_1_ and T_2_-weighted (T_2_w) sagittal spine imaging but does not mandate a complete set of imaging protocol parameters (the MY-RADS acquisition recommendations are summarised in Supplementary Table 1). Imaging sites are therefore required to optimise acquisition for their particular hardware and software in order to achieve high quality imaging.

Quantitative measurements of apparent diffusion coefficient (ADC) and fat fraction from WB-MRI show promise for evaluating and predicting treatment response [5–7]. By establishing acquisition protocols at a range of sites, multi-centre imaging studies are a crucial step in the translation of quantitative MR imaging biomarkers (qMR IBs) from research to clinical practice [8].

The feasibility of multi-centre WB-MRI has been demonstrated in healthy volunteers [9, 10] and, across a small number of sites, in patients with lymphoma [11, 12] and patients with myeloma [13]. Larger multi-centre WB-MRI studies have utilised imaging hubs, with patients referred to specialist imaging sites for scanning [14, 15]. This study is the first to establish standardised WB-MRI protocols across sites that reflect the variation in scanners and experience found in clinical practice and it is essential to evaluate the achievable image quality in this setting.

The purpose of this work was to evaluate the image quality achieved in a multi-centre WB-MRI study using the MY-RADS protocol across a range of scanner manufacturers and field strengths. Images were assessed qualitatively by radiological scoring and quantitatively using metrics developed to measure the presence and severity of image quality issues that frequently affect WB-MRI. The correlation between qualitative and quantitative metrics was evaluated, with a view towards developing tools for automated quality control (QC) of WB images in multi-centre studies.

## Methods

OPTIMUM/MUKnine (ClinicalTrials.gov Identifier: NCT03188172 [16]) is a prospective phase II study to screen for high-risk multiple myeloma [17, 18] and evaluate a novel treatment strategy. A sub-study of MUKnine, IMAGIng Minimal residual disease in Myeloma (IMAGIMM), is investigating the potential of WB-MRI to monitor treatment response in patients with multiple myeloma.

Patients enrolled in this sub-study underwent WB-MRI scans at three timepoints: baseline/study enrolment, 3 months post-autologous stem cell transplantation (ASCT) and 18–21 months post-ASCT. This evaluation included images from 121 WB-MRI examinations (from 83 individual patients across all timepoints) acquired for the OPTIMUM/MUKnine trial IMAGIMM sub-study across ten UK sites. This comprises all imaging data uploaded to the trial’s central imaging repository by 20 May 2022.

The sites underwent a site qualification process [19] to establish a MY-RADS-compliant imaging protocol consisting of axial DWI, axial T_1_w Dixon imaging and sagittal T_1_w and T_2_w spine imaging on a local scanner. Hardware and software limitations and scan time constraints required some protocol modifications between sites (full details are included in a prior publication [19]). Volunteer or exemplar patient data from each site were reviewed by the lead site to confirm that sufficient data quality was achievable prior to patient enrolment. Twelve sites were set up for the study; however, only ten went on to acquire patient data.

The scanners used for acquisition included five models from three manufacturers: 1.5 T MAGNETOM Aera, 1.5 T MAGNETOM Avanto, 3 T MAGNETOM Skyra (all Siemens Healthcare, Erlangen, Germany), 3 T Discovery MR750w (GE Healthcare, Milwaukee, USA) and 1.5 T and 3 T Ingenia (Philips Healthcare, Best, Netherlands). There were 110 examinations conducted at 1.5 T and 11 examinations conducted at 3 T. All data were sent to a central imaging repository at the lead site for QC and analysis.

Quantitative metrics are a valuable method for monitoring objective image quality; however, they must be linked to clinically relevant quality assessments. The following were identified as image artefacts or quality issues that commonly affect the quality of WB-MRI or DWI [20–22]:

1. Low signal to noise ratio (SNR)
2. Anterior thoracic signal loss
3. Susceptibility artefacts
4. Poor fat suppression
5. Ghosting
6. Geometric distortion
7. Eddy current distortion
8. Fat/water swaps

Each examination was scored both qualitatively and quantitatively as follows:

### Qualitative assessment

A radiologist with over 4 years of WB-MRI experience used a Likert scale, defined in Table 1, to rate the quality of the overall examination and each image series: DWI (focusing on images with *b*-values of 50 smm^−2^ (b50) and 900 smm^−2^ (b900), and ADC maps), Dixon (focusing on water and fat images) and spine imaging (T_1_w and T_2_w spine images were evaluated together and are referred to collectively as “spine imaging” in this work). The presence and severity of each of the eight artefacts/image quality issues described above was also evaluated.

**Table 1:** Likert scales used to score image quality and the presence/effect on diagnostic quality of each artefact/image quality issue

**Image quality**		**Presence/severity of artefacts—effect on diagnostic quality**	
**1**	Excellent	1	Not present/no artefact
**2**	Good	2	Minimal effect
**3**	Suboptimal	3	Moderate effect
**4**	Non-diagnostic	4	Severe effect

Susceptibility artefacts and fat/water swaps were scored for each artefact identified rather than for the whole examination. To capture regional variations, ghosting and geometric distortion were scored separately at the level of the pelvis and the brain. Differences in qualitative scores were evaluated for field strength (1.5 vs 3 T) and site using the Kruskal–Wallis *H* test.

### Quantitative assessment

**Fig. 1:**
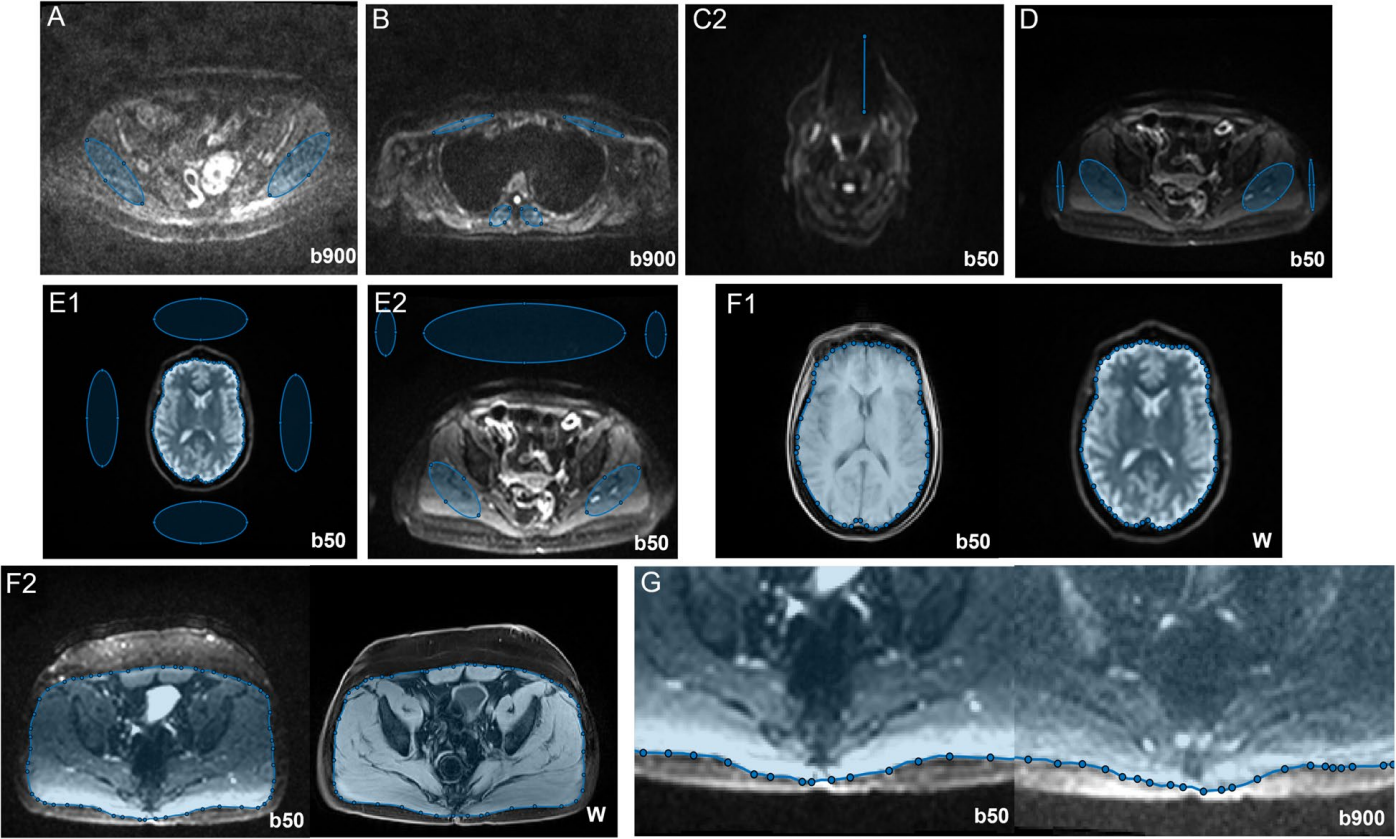
Examples of the method for calculating the quantitative metrics. The metrics for each artefact/image quality issue are defined in Table 2. The size of the ROIs varied between patients in accordance with anatomical differences. Metrics: A—signal to noise ratio; B—anterior thoracic signal loss; C2—susceptibility artefact, length; D—fat suppression; E1—ghosting (brain); E2—ghosting (pelvis); F1—geometric distortion (brain); F2—geometric distortion (pelvis); G—eddy current distortion. Image series: b50—DWI with *b*-value = 50 smm^−2^; b900—DWI with *b*-value = 900 smm^−2^; W—Dixon water image

A semi-automated pipeline was developed in Matlab (R2019a, MathWorks, Natick, MA, USA) to calculate metrics related to each of the eight artefacts/image quality issues. Each quantitative metric is described in Table 2, with examples provided in Fig. 1. These metrics were developed in collaboration with a radiologist, with the intention that they should relate to clinically relevant features.

**Table 2:** Each of the image quality issues/artefacts is defined in terms of the image series and location defined, and the calculation of quantitative metric

	**Artefact/image quality issue**	**Slice location**	**Image series**	**Description**	**Metric**
**A**	Signal to noise ratio (SNR)	Pelvic	DWI – b900	Bilateral ROIs were defined over the gluteal muscle.	$\frac{std(gluteal\ signal)}{mean(gluteal\ signal)}$
**B**	Anterior thoracic signal loss	Thoracic	DWI – b900	Bilateral ROIs were defined over the pectoral and paravertebral muscle.	$\frac{mean(paravertebral\ signal)}{mean(pectoral\ signal)}$
**C**	Metal susceptibility artefacts	Anywhere	DWI – b50	The radiologist identified the location. The number of affected slices was observed manually and a measurement tool was used to measure the maximum extent in the A/P direction.	**C1:*** No. affected slices* **C2:*** Maximum extent in A/P direction (mm)*
**D**	Fat suppression	Pelvic	DWI – b50	Bilateral ROIs are defined over the gluteal muscle and over the adjacent fat.	$\frac{mean(fat\ signal)}{mean(gluteal\ signal)}$
**E**	Ghosting	**E1:** Brain	DWI – b50	A contour was defined around the surface of the brain and four ROIs were defined in the background (anterior, posterior, left and right).	100*$$\frac{\begin{array}{c}\left(top\ bg+bottom\ bg\right)-\\ (left\ bg+right\ bg)\end{array}}{2\ mean(brain\ signal)}$$
		**E2:** Pelvic	DWI – b50	Bilateral ROIs were defined over the gluteal muscle and three ROIs were defined in the background (anterior and in the top corners).	100*$\frac{2\left(top\ bg\right)-(left\ bg+right\ bg)}{2\ mean(gluteal\ signal)}$
**F**	Geometric distortion	**F1:** Brain	DWI – b50 Dixon (water)	A contour was defined around the surface of the brain on both series	*Hausdorff distance between the two contours*
		**F2:** Pelvic	DWI – b50 Dixon (water)	A contour was defined around the surface of the muscle on both series	*Hausdorff distance between the two contours*
**G**	Eddy current distortion	Pelvic	DWI – b50 DWI – b900	A contour was defined around the surface of the muscle on both series. The anterior half of the image was discarded to exclude the effect of respiratory motion and the laterally interior 30 cm region was used to exclude the difficult-to-define lateral regions.	*Hausdorff distance between the two contours*
**H**	Fat/water swaps	Anywhere	Dixon (water)	The radiologist identified the location.	*No quantitative metric*

Three slice locations were identified for measurements:

- Pelvis—at the widest point of the gluteal muscle on the axial cross-section
- Thorax—at the widest point of the pectoral muscle on the axial cross-section
- Brain—immediately superior to the orbits

For most metrics, measurements were made at one of these locations, chosen as the location where it was most suitable to measure. For each metric, the same location was used for all examinations. The physicist was required to identify the station and slice numbers corresponding to these locations, and to define the ROIs.

Measurements were made on the image series where the issue is likely to be most significant, e.g. SNR measurements were made on the b900 DW image as signal is inherently low. Some metrics were comparative, e.g. distortion on a b50 DW image is measured by comparing a contour to the equivalent contour in the water-only Dixon series.

Susceptibility artefacts can occur at any location and were therefore identified by the radiologist and measurements made wherever they occurred. No quantitative measure was developed for fat/water swaps as these are either present or not present. Examinations were grouped according to the qualitative score they received for each issue/artefact and one-way ANOVAs with Tukey post hoc tests were used to assess for group differences in quantitative scores.

Ordinal logistic regression was used to create a model of the relationship between all the quantitative metrics and the radiological score for DWI quality. The quantitative scores were prepared for this analysis as follows:

- The natural logarithm was taken for any ratio metric (e.g. SNR or fat suppression) to linearise the response [23].
- The reciprocal of ln(SNR) was taken so that a higher score corresponds to lower quality for all metrics.
- Both susceptibility artefact metrics were aggregated across multiple artefacts to give total number of slices and total length as predictor variables.
- All metrics were normalised onto an equivalent scale by calculating the mean and standard deviation across all examinations, then for each score subtracting the mean and dividing by the standard deviation.

### Repeatability/reproducibility

Ten examinations, one from each site, were randomly selected for a sub-study to assess the repeatability of scoring. To examine intra-rater repeatability, the same radiologist repeated the qualitative scoring and the same physicist repeated the quantitative scoring. For inter-rater repeatability, a different radiologist (with 3 years of experience reporting WB-MRI) repeated the qualitative scoring and a different physicist repeated the quantitative scoring for the same subset of ten examinations.

Cohen’s weighted kappa, using the categories of agreement proposed by Landis and Koch [24], was used to assess the significance of intra- and inter-rater differences for the qualitative measures. The repeatability of quantitative scoring was assessed with Bland–Altman analysis and the intraclass correlation coefficient (ICC).

The difference between an “excellent” and “good” examination is unlikely to be as clinically significant as the difference between a “good” and “suboptimal” examination. The qualitative scores were therefore binarised into two categories, excellent/good and sub-optimal/non-diagnostic, and assessed in terms of percentage agreement.

## Results

### Qualitative assessment

**Fig. 2:**
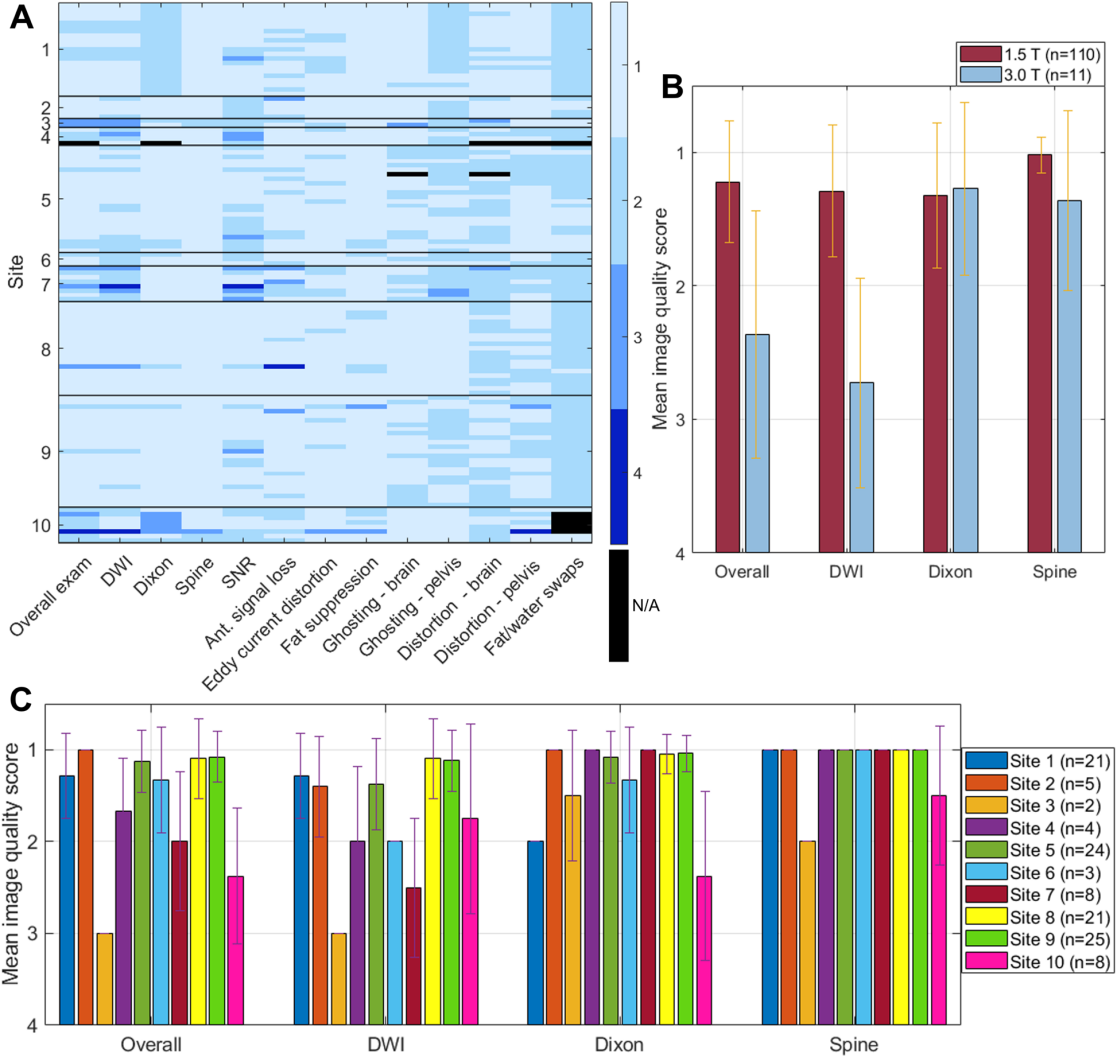
Summary of qualitative image scoring. **A** Representation of qualitative scores for both image quality and artefact presence/severity across all examinations. Each row represents a single examination, with examinations grouped according to site. Each column represents a different scoring metric. A black rectangle indicates that a score was not possible for that examination, e.g. Dixon imaging could not be scored because it was not provided, or brain distortion could not be scored as the first imaging station was not acquired due to patient kyphosis. **B**, **C** Image quality scores separated by field strength and site respectively. The dashed braces in **A** indicate groups for which a statistically significant difference in means was found, using a Mann–Whitney *U* test

**Fig. 3:**
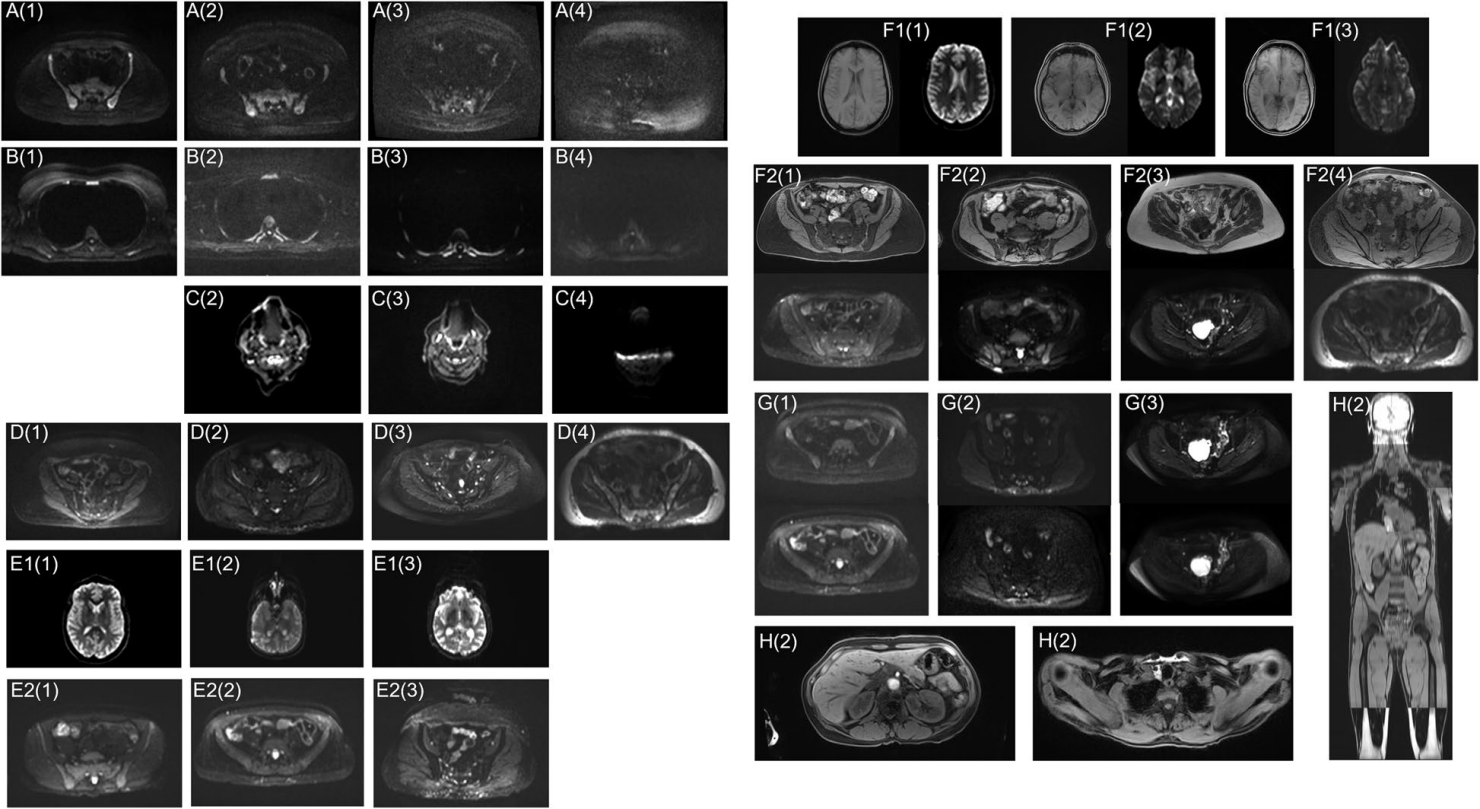
Examples of each artefact/image quality issue that received each score for presence/severity. Artefacts/quality issues are identified by the letters given in Table 2 and scores are indicated by the numbers in brackets (according to the Likert scale: 1 = not present/no artefact, 2 = minimal effect, 3 = moderate effect, 4 = severe effect). When a score is not shown for a particular artefact, this indicates that no examinations were given this score. Images are windowed by a radiologist to optimise reading for each series

Qualitative scoring for image quality and artefact presence/severity is summarised in Table 3 and Fig. 2, with examples of each score provided in Fig. 3.

**Table 3:** The number of examinations receiving each image quality score for diffusion-weighted imaging (DWI), Dixon imaging, sagittal spine imaging and overall examination. Note that Dixon imaging was not provided for one examination. To maintain consistency in the definition of overall exam this exam was excluded from the overall scoring, although Dixon and spine imaging were scored

	**Image quality score (number of exams)**				
	**1—excellent**	**2—good**	**3—suboptimal**	**4—non-diagnostic**	**Total**
**DWI**	80	33	6	2	121
**Dixon**	87	28	5	0	120
**Spine**	116	4	1	0	121
**Overall exam**	89	24	6	1	120

94.2% of examinations received a score of either good or excellent for overall image quality, with 93.4%, 95.8% and 99.2% receiving good or excellent scores for DWI, Dixon and spine imaging, respectively. This reflects that DWI generally remains marginally more challenging to implement than the rest of the protocol, although 66.1% of DWI exams were rated as excellent with only two (1.7%) rated as non-diagnostic.

A Kruskal–Wallis *H* test determined that the qualitative scores at 1.5 T were significantly higher than those at 3 T for overall exams (*χ*^2^(1) = 24.6,* p* < 0.001), DWI (*χ*^2^(1) = 32.0,* p* < 0.001) and spine imaging (*χ*^2^(1) = 16.4, *p* < 0.001), with no statistically significant difference for Dixon imaging (*χ*^2^(1) = 0.6, *p* = 0.559).

A Kruskal–Wallis *H* test showed a statistically significant difference in mean score between at least two sites for the overall exams (*χ*^2^(9) = 57.5, *p* < 0.001), DWI (*χ*^2^(9) = 47.4, *p* < 0.001), Dixon (*χ*^2^(9) = 86.2, *p* < 0.001) and spine imaging (*χ*^2^(9) = 72.5, *p* < 0.001).

### Repeatability/reproducibility—qualitative scores

**Fig. 4:**
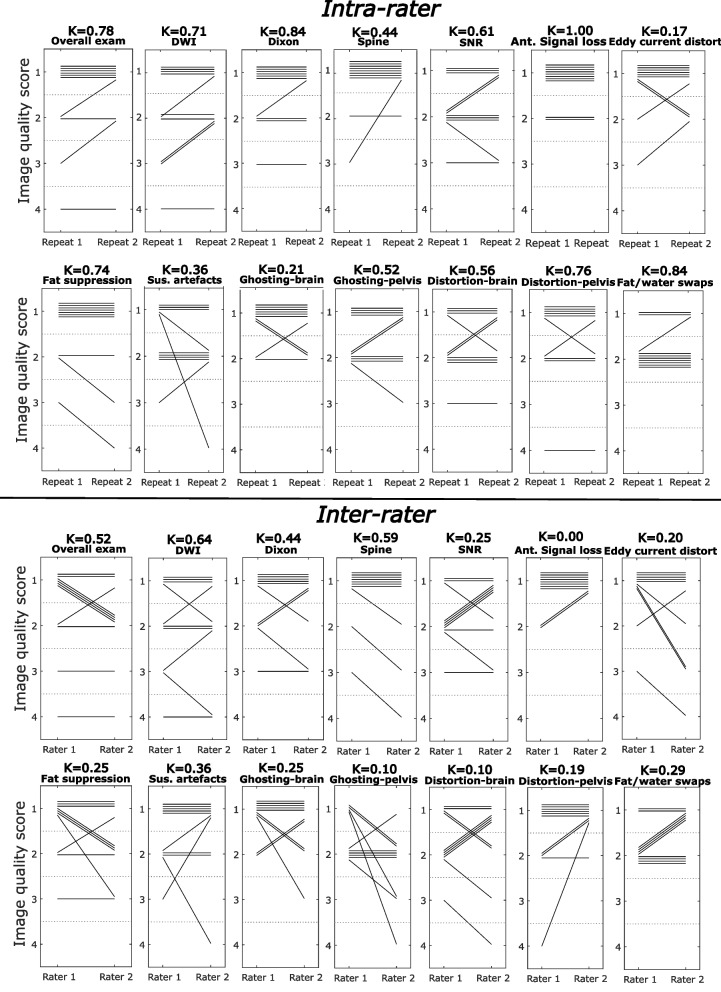
Intra- and inter-rater repeatability of qualitative scoring. Plots illustrating the intra-rater and inter-rater agreement for each image quality and artefact scoring across a subset of patients. Each line represents an individual patient so that a horizontal line indicates that the same score was given in both assessments. For each plot the Cohen’s weighted kappa coefficient is displayed with 95% confidence intervals

Intra- and inter-rater repeatability is illustrated graphically in Fig. 4. For the intra-rater image scoring, the agreement was excellent for Dixon imaging, substantial for overall exams and DWI and moderate for spine imaging. For the artefact scoring, the agreement was moderate or higher for all metrics apart from susceptibility artefacts, brain ghosting and eddy current distortion.

For the inter-rater image scoring, the agreement was substantial for DWI and moderate for overall exams, Dixon imaging and spine imaging. For the artefact scoring, the agreement was fair for all metrics except brain distortion, anterior signal loss, brain ghosting and pelvic ghosting, for which it was slight/poor.

When scores were binarised into excellent/good and sub-optimal/non-diagnostic categories, all scores had an intra-rater percentage agreement of between 80 and 100% and an inter-rater percentage agreement of between 70 and 100%.

### Quantitative assessment

**Fig. 5:**
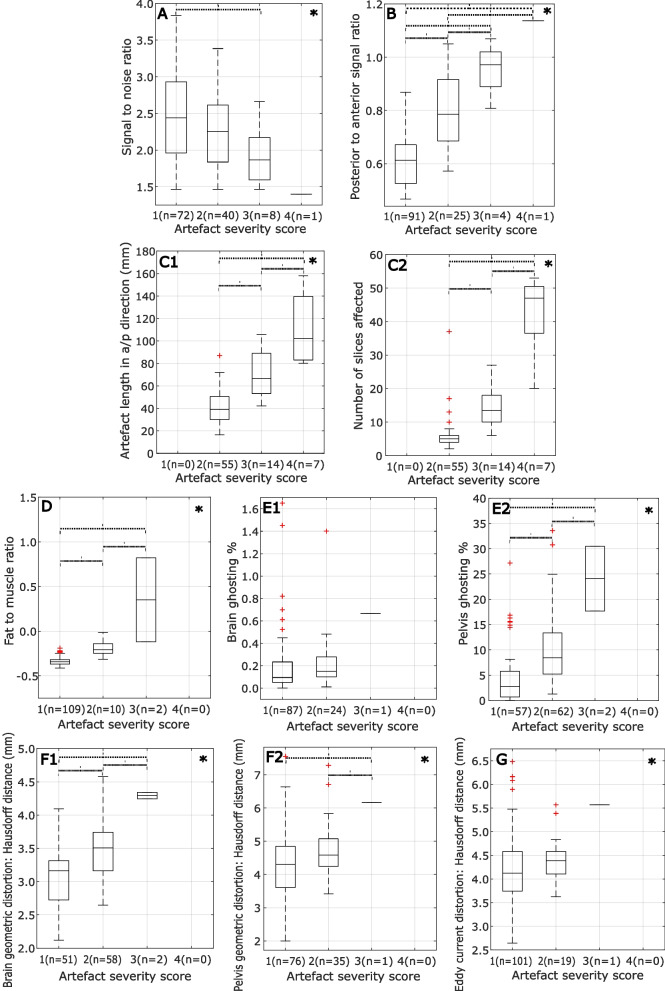
Correlation between qualitative and quantitative scoring metrics. Boxplots illustrating the quantitative measures for each artefact/image quality issue, grouped according to qualitative score. An asterisk in the top-right of a plot indicates that a statistically significant group difference was found for that metric using a one-way ANOVA. Significant differences between individual groups, as determined using Tukey’s HSD test for multiple comparisons, are indicated by the dashed braces

Figure 5 illustrates the quantitative scoring, with examinations grouped by their qualitative scores.

A one-way ANOVA found a statistically significant group difference in quantitative score between at least two groups for the following metrics: SNR (*F*(3,117) = 3.50, *p* = 0.018), anterior thoracic signal loss (*F*(3,117) = 41.71, *p* < 0.001), susceptibility number of affected slices (*F*(2,73) = 112.14, *p* < 0.001), susceptibility length (*F*(2,73) = 59.06, *p* < 0.001), fat suppression (*F*(2,118) = 89.77,* p* < 0.001), pelvic ghosting (*F*(2,118) = 19.67, *p* < 0.001) and brain geometric distortion (*F*(2,108) = 19.20, *p* < 0.007). Tukey’s HSD test for multiple comparisons was used to compare scores between individual groups, as indicated in Fig. 5. There was no statistically significant group difference for brain ghosting (*p* = 0.156) or eddy current distortion (*p* = 0.108).

The results of the ordinal logistic regression model are summarised in Table 4. The normalised metrics for SNR, anterior signal loss and brain distortion were found to be statistically significant predictors of DWI image quality.

**Table 4:** The model was used to predict the radiological DWI scan quality using all ten quantitative metrics. Metrics that were found to be statistically significant predictors are indicated with an asterisk

**Metric**		**Coefficient (*β*)**	***p*-value**	**Odds ratio**	**Odds ratio 95% CI**
SNR	*	− 0.483	.032	0.62	0.40–0.96
Anterior thoracic signal loss	*	− 0.716	.002	0.49	0.31–0.76
Susceptibility artefact—total no. slices		0.311	.490	1.36	0.56–3.31
Susceptibility artefact—total length		− 0.616	.176	0.54	0.22–1.32
Eddy current distortion		0.357	.148	1.43	0.88–2.32
Fat suppression		− 0.329	.132	0.72	0.47–1.10
Ghosting—brain		− 0.469	.060	0.63	0.38–1.02
Ghosting—pelvis		− 0.457	.087	0.63	0.38–1.07
Distortion—brain	*	− 0.536	.019	0.59	0.37–0.92
Distortion—pelvis		− 0.222	.341	0.80	0.51–1.26

*Metrics that were found to be statistically significant predictors are indicated with an asterisk

The odds of an exam receiving a higher quality score were reduced by a factor of 0.62 (95% CI: 0.40–0.96), 0.49 (95% CI: 0.31–0.76) and 0.59 (95% CI: 0.37–0.92) for a unit increase in the normalised measures of 1/SNR, anterior signal loss and brain distortion, respectively.

### Repeatability/reproducibility—quantitative scores

**Fig. 6:**
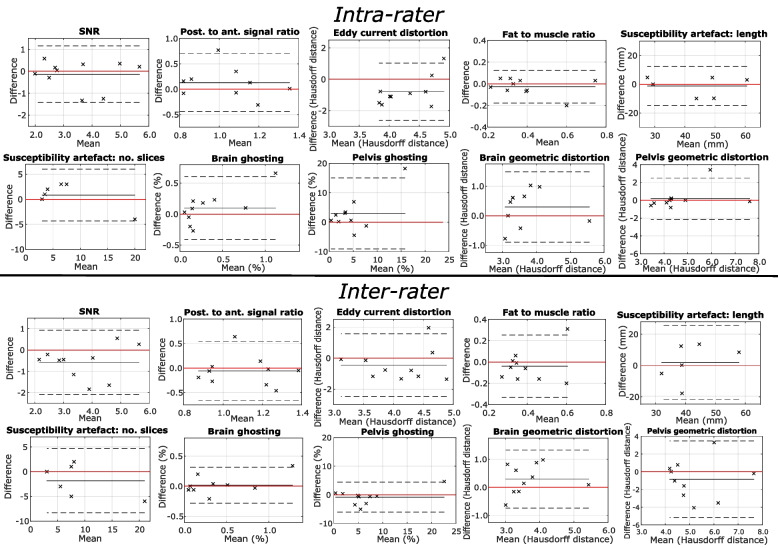
Repeatability of quantitative scoring. Bland Altman plots illustrating the intra-rater and inter-rater agreement for each artefact/image quality issue. In each case, the mean difference is plotted with a solid black line and zero difference is plotted with a solid red line. The dotted lines indicate the 95% confidence limits (mean difference ± 2*std.)

The repeatability of the quantitative scoring is summarised in Table 5, with Bland–Altman plots presented in Fig. 6.

**Table 5:** Summary of the intra- and interrater correlation of quality scores for each quantitative metric. Metrics are identified according to the letters assigned in Table 2

Intra-rater										
**Metric**	**A**	**B**	**C1**	**C2**	**D**	**E1**	**E2**	**F1**	**F2**	**G**
**ICC**	0.91	0.17	0.95	0.88	0.83	0.74	0.32	0.59	0.66	0.07
***p***	< .001	.282	< .001	.006	< .001	.004	.138	.019	.016	.372
**Mean bias**	− 0.06	0.16	0.83	− 1.23	− 0.05	0.10	3.29	0.38	0.27	− 0.80
Inter-rater										
**Metric**	**A**	**B**	**C1**	**C2**	**D**	**E1**	**E2**	**F1**	**F2**	**G**
**ICC**	0.51	0.01	0.85	0.40	0.36	0.92	0.90	0.65	0.08	0.08
***p***	.032	.489	.005	.211	.131	< .001	< .001	.007	.413	.413
**Mean bias**	− 0.77	− 0.03	− 1.83	1.98	− 0.06	0.00	− 0.52	0.37	− 0.65	− 0.65

*A* SNR *B* anterior thoracic signal loss, *C1* susceptibility: number of slices, *C2* susceptibility: length, *D* fat suppression, *E1* ghosting (brain), *E2* ghosting (pelvis), *F1* geometric distortion (brain), *F2* geometric distortion (pelvis), *G* eddy current distortion

For the intra-rater comparison, ICC was found to be higher than 0.75 (considered to indicate good reliability [25]) and statistically significant (Bonferroni-corrected *α* = 0.005) for SNR (ICC = 0.91, *p* < 0.001), fat suppression (ICC = 0.83, *p* < 0.001), brain ghosting (ICC = 0.74, *p* = 0.004) and susceptibility artefact number of slices (ICC = 0.95,* p* < 0.001). For the inter-rater comparison, this was the case for SNR (ICC = 0.51, *p* = 0.032), brain ghosting (ICC = 0.92, *p* < 0.001), pelvic ghosting (ICC = 0.90, *p* < 0.001), brain distortion (ICC = 0.65, *p* < 0.007) and susceptibility artefact number of slices (ICC = 0.85, *p* = 0.005).

## Discussion

The MY-RADS guidelines promote standardisation for WB-MRI; however, image quality using the MY-RADS protocol has not previously been assessed in a large multi-centre study. For WB-MRI to become a widely available clinical tool outside of specialist centres, good image quality must be achievable across the range of hardware and software in use. Sites participating in the MUKnine IMAGIMM sub-study were invited based on their patient population and not prior WB-MRI experience, providing an opportunity to evaluate the achievable image quality in a realistic multi-centre WB-MRI study.

Out of 121 examinations from ten varied sites, 120 were judged by a radiologist to be diagnostic with 89 of those being of excellent overall quality. The high proportion of exams rated as good or excellent shows that the MY-RADS protocol can be successfully implemented in a representative patient cohort across a variety of sites. This result was achieved despite the additional challenges of the COVID-19 pandemic, which coincided with the study.

Only one overall examination was deemed to be non-diagnostic, scoring poorly across all series. In this case, the poor image quality can be linked to non-compliance with the desired imaging protocol, with DWI acquired with only two *b*-values, insufficient averaging and an incorrect slice thickness (6 mm rather than 5 mm). A different scanner was used to that which was qualified for this site, underlining the importance of the site qualification process for establishing protocols that deliver good image quality.

One other exam was reported to have non-diagnostic DW images. In this case, the examination was compliant with the imaging protocol; however, the quality of the b900 images was degraded by a loss of SNR due to the patient’s size and a substantial susceptibility artefact in the region of a metallic implant in the spine. The excellent quality of the Dixon and spine imaging meant that the overall exam retained some diagnostic value.

The qualitative radiological image scoring found that overall exams, DWI and spine imaging are higher quality at 1.5 T than at 3 T. The degree of anterior thoracic signal loss and geometric distortion at 3 T suggests that there are still challenges related to B_0_ field homogeneity in the implementation of standardised protocols across the fleet of available scanners.

There were some limitations to this study, including the uneven distribution of manufacturer and field strength. 111 examinations were from a single manufacturer and 110 were conducted at 1.5 T, making it difficult to separate manufacturer, field strength and site-specific performance. No inferences have therefore been drawn regarding image quality across different scanner manufacturers. The quantitative measurements are limited by their reliance on a single imaging slice and therefore do not reflect the potential inhomogeneity of effects.

Both qualitative and quantitative scoring have a degree of subjectivity and repeatability must be assessed; however, Cohen’s kappa can be misleadingly low for small sample sizes such as this. For example, the inter-rater percentage agreement for qualitative anterior signal loss was 80%; however, the distribution of scores for this metric meant that Cohen’s kappa was 0.00, implying very poor agreement. Figure 4 demonstrates visually that the intra- and inter-rater repeatability between qualitative scores was generally good, providing reassurance that the radiological image scoring is a relatively objective measure of clinical image quality.

The quantitative metrics need to demonstrate significant correlation with a radiological assessment of clinical significance. This was the case for several of the metrics defined here, including SNR, anterior signal loss and the measures of susceptibility artefacts. Some metrics, such as fat/water swaps, were relatively common but generally did not affect diagnostic quality while others, such as eddy current distortion, occurred very rarely in these examinations. Clinical outcome was not considered in this work; however, it is assumed that radiological image quality is associated with lesion detection.

Manual assessment of image quality is time-consuming and impractical for larger cohorts so there is potential value in the development of automated quality assessment pipelines that reflect clinical interpretation of quality [26, 27]. When the overall quality of a WB-MRI examination was sub-optimal or non-diagnostic in this dataset, this was likely to be because of DWI quality issues. SNR, anterior/posterior signal ratio and brain distortion measurements were found to be statistically significant predictors of DWI quality and could therefore form an automated pipeline to predict radiological image quality. Retrospectively, this could be used to rapidly highlight sites providing poor quality imaging so that underlying issues can be addressed. An automated pipeline could also be implemented prospectively during protocol development or routine clinical scanning providing the user with feedback on quality that informs protocol development or modification.

The SNR of b900 DW images correlates with radiological assessment of SNR, is a significant predictor of qualitative image quality and demonstrates good repeatability. It is therefore proposed that the SNR of b900 DW images is the most important factor determining the quality of WB-MRI examinations and that measurement of SNR may be used to predict exam quality. The use of simple SNR measurements should be investigated further to characterise the performance of a particular scanner or acquisition sequence for WB-DWI and to provide a benchmark for acceptable image quality in multi-centre trials.

## Conclusions

This image quality assessment has shown for the first time that it is possible to successfully deliver a multi-centre WB-MRI study using the MY-RADS protocol, even from sites with a range of hardware and prior WB-MRI experience. This underlines the importance of the site qualification process [19], which established acquisition protocols that were optimised to local conditions and ensured that all sites were capable of delivering high quality imaging prior to patient enrolment. Quantitative metrics of image quality have been shown to have good repeatability and correlation with radiological assessment and could be developed further to provide a pipeline for automated QC of WB-MRI data in multi-centre studies.

### Supplementary Information

**Additional file 1: Supplementary Table 1. **The MY-RADS recommended protocol for WB-MRI.

## Data Availability

Due to privacy regulations, the data used in this study are not publicly available. In order to see and discuss the data, the authors can be contacted. If needed, we can arrange approval to share the data with individual researchers.

## Abbreviations

ADC: Apparent diffusion coefficient
ASCT: Autologous stem cell transplantation
DWI: Diffusion weighted imaging
EPI: Echo planar imaging
ICC: Intraclass correlation coefficient
MY-RADS: Myeloma Response Assessment and Diagnosis System
PET/CT: Positron emission tomography/computed tomography
QC: Quality control
qMR IBs: Quantitative magnetic resonance imaging biomarkers
ROI: Region of interest
SNR: Signal to noise ratio
WB-MRI: Whole-body magnetic resonance imaging
