# Supplementary material for: Image quality in whole-body MRI using the MY-RADS protocol in a prospective multi-centre multiple myeloma study
Source: Insights Imaging. 2023 Oct 15;14:170. doi: 10.1186/s13244-023-01498-3 (PMC10577121; doi:10.1186/s13244-023-01498-3)
Supplement: Supplementary file 1 — Additional file 1: Supplementary Table 1. The MY-RADS recommended protocol for WB-MRI. [file 13244_2023_1498_MOESM1_ESM.docx]

**Image quality in whole-body MRI using the MY-RADS protocol in a prospective multi-centre multiple myeloma study**

**ELECTRONIC SUPPLEMENTARY MATERIAL**

**Supplementary Table 1:** The MY-RADS recommended protocol for WB-MRI.

| Sequence | Description | | Core clinical protocol | Comprehensive research protocol |
| --- | --- | --- | --- | --- |
| T_1_w spine | Sagittal, whole spine, T_1_-weighted fast spin echo. 4-5 mm slices. | | Yes | Yes |
| T_2_w spine | Sagittal, whole spine, STIR or fat-suppressed T_2_-weighted. 4-5 mm slices. | | Yes | Yes |
| Dixon | Axial or coronal, whole body (vertex to knees), gradient echo Dixon. 5 mm slices. | | Yes  (Axial or coronal) | Yes  (Axial and coronal) |
|  |  | Fat and water image reconstructions |  |  |
|  |  | Fat fraction map |  |  |
| DWI | Axial, whole body (vertex to knees), DWI with STIR fat suppression. 5 mm slices. | | Yes  2 b-values (50-100 smm^-2^ and 800-900 smm^-2^) | Yes  3 b-values (additional 500-600 smm^-2^) |
|  |  | ADC map |  |  |
|  |  | 3D MIP with highest b-value |  |  |
| T_2_w whole body | Axial, whole body (vertex to knees), T_2_-weighted fast spin echo without fat suppression (preferably matching the DWI). 5 mm slices. | | Optional | Yes |
| Regional assessment | Additional regional assessment for symptomatic or known sites outside of standard FOV. | | Usually not | Optional |

**Supplementary Table 1:** Summary of the recommended MY-RADS protocol for WB-MRI in patients with myeloma. This table is based on the table presented in Messiou *et al* (4).
